# Inflammatory Skin Lesions in Three SARS-CoV-2 Swab-Negative Adolescents: A Possible COVID-19 Sneaky Manifestation?

**DOI:** 10.3390/pediatric13020025

**Published:** 2021-04-09

**Authors:** Giuseppe Ingravallo, Francesco Mazzotta, Leonardo Resta, Sara Sablone, Gerardo Cazzato, Antonietta Cimmino, Roberta Rossi, Anna Colagrande, Beniamino Ferrante, Teresa Troccoli, Ernesto Bonifazi

**Affiliations:** 1 Department of Emergency and Organ Transplantation, Section of Pathology, University of Bari Aldo Moro, 11, 70124 Bari, Italy; leonardo.resta@uniba.it (L.R.); gerycazzato@hotmail.it (G.C.); micasucci@inwind.it (A.C.); roberta.rossi300675@libero.it (R.R.); anna.colagrande@gmail.com (A.C.); 2 Pediatric Dermatology and Surgery Outpatients Department, Azienda Sanitaria Locale Barletta-Andria-Trani, 76123 Andria, Italy; cetromazderm@libero.it (F.M.); beniaminoferrante@gmail.com (B.F.); teresatroccoli63@gmail.com (T.T.); 3 Section of Forensic Medicine, Department of Interdisciplinary Medicine, University of Bari Aldo Moro, 70124 Bari, Italy; sarasabloneml@gmail.com; 4 Pediatric Dermatology, University of Bari Aldo Moro, 11, 70124 Bari, Italy; ejpd@dermatologiapediatrica.com

**Keywords:** SARS-CoV-2, skin, granuloma annulare, chilblains

## Abstract

Coronavirus disease 19 (COVID-19), caused by severe acute respiratory syndrome coronavirus 2 (SARS-CoV-2), is associated with various clinical manifestations, including skin lesions. In particular, during the COVID-19 pandemic lock-down period numerous chilblain-like lesions, mainly located on the feet, were observed in adolescents. The latter were often asymptomatic or associated with very mild respiratory symptoms. Here, we report three cases of acral nodular lesions in SARS-CoV-2 swab-negative adolescents with histological findings of chronic immune-mediated inflammation and immunohistochemical evidence of SARS-CoV-2 spike glycoproteins in endothelial cells and eccrine sweat glands. In one of these cases, the virus presence was confirmed by electron microscopy.

## 1. Introduction

During the COVID-19 pandemic, increasing numbers of skin lesions have been described in positive individuals [1,2,3,4]. Various reports [1,2,3,4,5,6] have offered clinical documentation of cutaneous lesions in adults, such as Erythema Pernio, Erythema Multiforme, Chilblains, Urticated Erythema, Morbilliform, Varicelliform, Chickenpox-like exanthem, etc. In the pediatric population, the incidence of skin manifestations reported by various authors is low: 0.25% [7,8]. Biopsies from skin lesions in children with confirmed or suspected COVID-19 have rarely been described in the literature [8]. Most of the histopathological descriptions were of isolated cases or small series, and some types of lesions have been biopsied only in adults. In general, the histopathological patterns were similar to chilblains [9,10], maculopapular eruptions [11,12], Erythema Multiforme [13,14], and purpuric and livedoid forms [15,16,17].

In this brief article, we present three cases of SARS-CoV-2 swab-negative adolescents, with no flu-like symptoms in the previous months, who developed nodular lesions of the feet reminiscent of deep granuloma annulare. A thorough histopathological analysis was carried out in these cases.

## 2. Case Presentation

**Figure 1:**
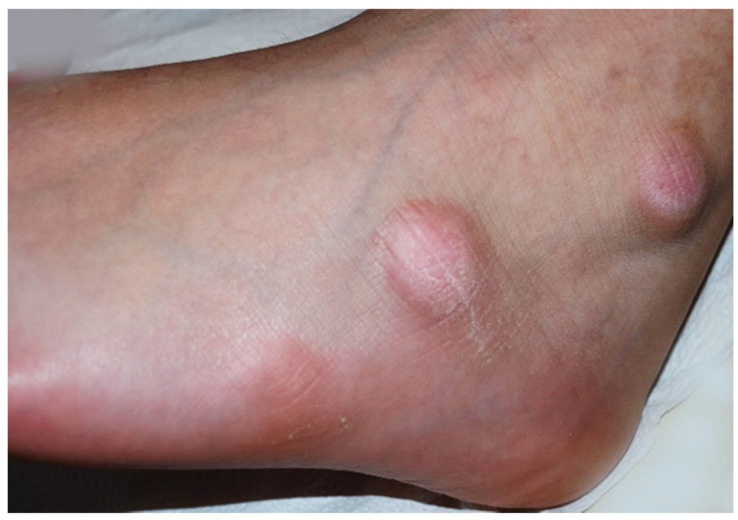
Asymptomatic firm nodules measuring 2–3 cm in diameter on the lateral aspect of the left foot.

**Figure 2:**
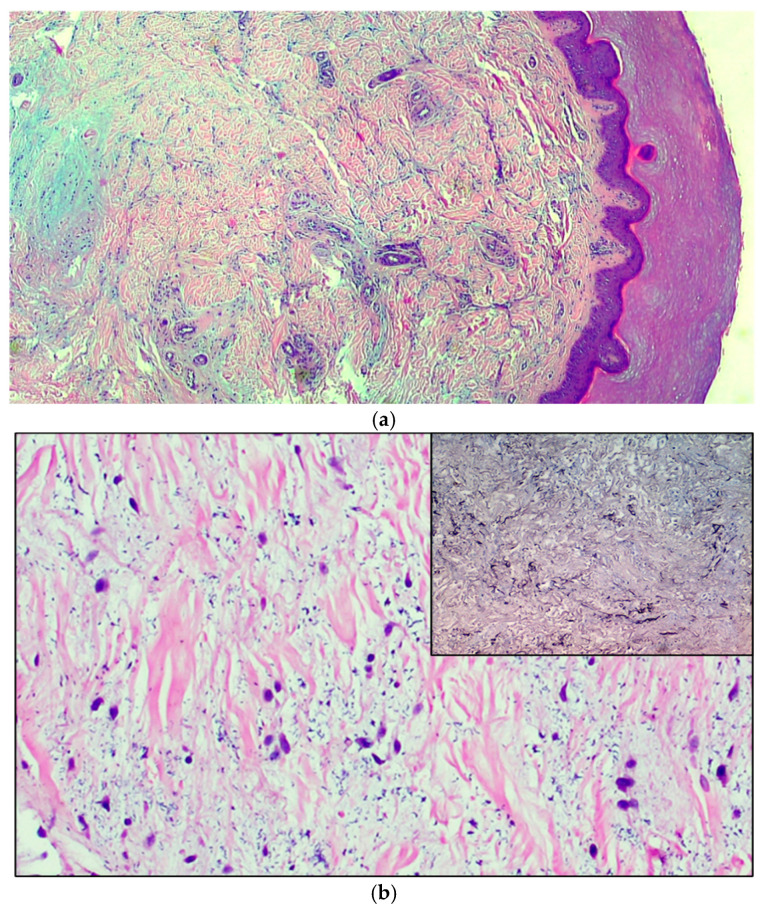

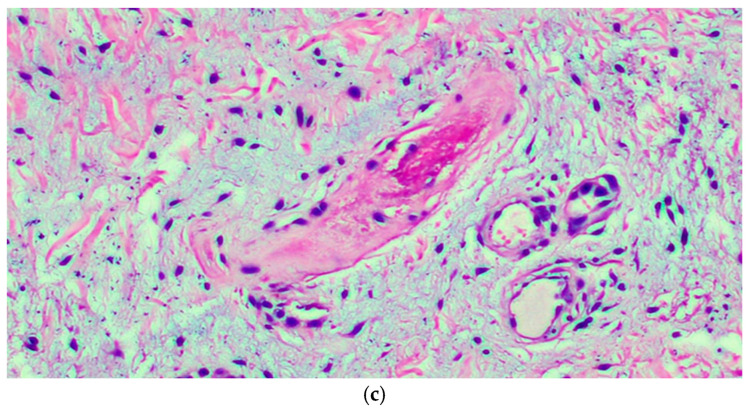
(**a**) The microphotograph shows orthokeratosis and mild acanthosis (acral skin), a mild lymphomonocyte infiltrate and myxoid stroma of the dermis. Hematoxylin-Eosin staining, original magnification 100×. (**b**) In the medial deep dermis, an ample area of edema is evident, with extensive and accentuated fragmentation or even dissolution of the collagen and elastic fibers (the latter confirmed by the Orceina staining), together with the presence of thrombotic foci in the small vessels. (Hematoxylin-Eosin staining, original magnification, 400×, with orcein staining in the insert). (**c**) Detail of a thrombosed blood vessel together with the presence of fibroblasts of a likely “reactive” significance around extensive, widespread dermal mucinosis. Hematoxylin-Eosin staining, original magnification 400×.

**Case 1:** An 11-year-old boy was observed for multiple swelling of the feet associated with mild burning. His family and personal history was negative for connective tissue, autoimmune or other noteworthy diseases. A physical examination showed 7–8 lesions (Figure 1) on the lateral surface of the feet, that were slightly erythematous and of a hard-elastic consistency. The nodules were not painful on palpation, mobile on the deep plane, with a diameter ranging between 6 and 12 mm, and had appeared about 20 days earlier. Due to the uncertain clinical features, we decided to perform a biopsy, after molecular testing for SARS-CoV-2 resulted negative. A histological examination (Figure 2a) showed acral skin with mild hypergranulosis, acanthosis, and focal vacuolization of the basal keratinocytes. Below the dermo-epidermal junction there was ectasia of the vessels of the superficial capillary plexus, that presented a narrow lumen with protruding endothelial cells (hobnail). Accentuated dermo-hypodermic mucinosis coexisted with fragmentation or even dissolution of the collagen and elastic fibers (Figure 2b). The nodules were treated with topical corticosteroids and subsided in about 4 months. A serological test carried out after 3 and a half months was positive for IgG.

**Case 2:** A 14-year-old boy, already known since he suffers from persistent atopic dermatitis, was observed during the pandemic due to exacerbation of his dermatitis. During the examination we observed the presence of asymptomatic nodules, clinically comparable to those of case 1, which had appeared about 20 days earlier.

Therefore, we performed a punch biopsy. A histological examination showed orthokeratosis and mild acanthosis. Immediately below the dermal-epidermal junction, the vessels of the superficial capillary plexus presented a narrow lumen with protruding endothelial cells (hobnail), sometimes completely occluding the vessel lumens. In the middle and deep dermis an ample area of edema, with extensive and marked fragmentation or even dissolution of the collagen and elastic fibers, was evident, as well as thrombotic foci (Figure 2c) of the small vessels, which showed sclero-hyalinosis of the wall and hemorrhagic suffusion. There were also fibroblasts of a likely reactive significance around extensive, widespread dermal mucinosis. A serological test carried out after 4 months was positive for IgG.

**Case 3:** An 11-year-old girl, with no notable medical history, developed acro-localized nodular lesions similar to those of cases 1 and 2 during the pandemic period. We again performed a punch biopsy. A histological examination showed acanthosis and mucinosis as in case 2. The vessels showed a narrow lumen with protruding endothelial cells and there was also sclero-hyalinosis of the wall and hemorrhagic suffusion.

**Methods.** The samples were fixed in 20% formalin for light microscopy and after processing, paraffin-embedding and microtome cutting, sections were prepared for routine staining with Hematoxylin-Eosin.

Then, 5 µm thick sections of all cases were prepared for immunostaining with anti-SARS-CoV-2 Spike glycoprotein S1 monoclonal antibody (ThermoFisher) for Coronavirus (MA5-36247), with 100 µg at 1 mg/mL concentration, isotype: IgG. Appropriate negative and positive controls were included in the procedure.

In addition, a small portion of the sample of only one case was post-fixed with 1% osmium tetroxide in PBS for 2 h at 4 °C for electronic microscopy. The fixed specimens were processed for embedding in Epoxy-Resin-Araldite (M) CY212 (TAAB, Aldermaston, UK). Semi-thin (two micron thick) sections were stained with toluidine blue. Ultrathin sections were mounted on formvar-coated nickel grids and routinely stained with uranyl acetate and lead citrate. Images of the semi-thin sections were captured using a Nikon photomicroscope equipped with a Nikon Digital sight DS-U1 camera (Nikon Instruments SpA, Calenzano, Italy). The ultra-thin sections were observed using a transmission electron microscope (Morgagni 268, FEI Company, Naples, Italy).

## 3. Results

**Figure 3:**
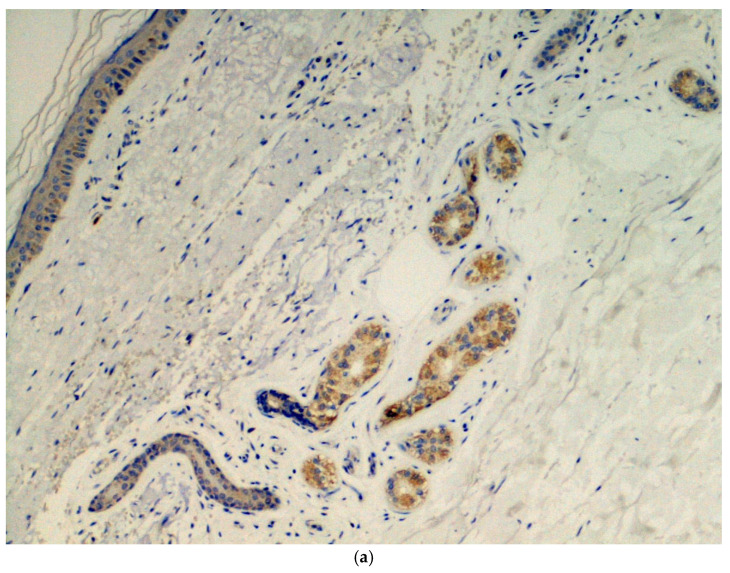

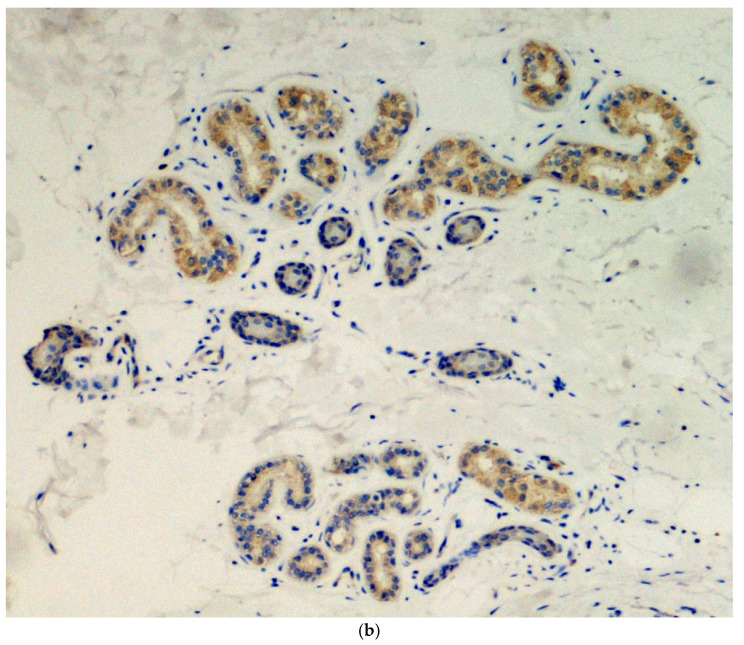
(**a**) Presence of viral spike proteins in the cytoplasm of epithelial cells of the secretory portion of eccrine sweat glands (brown color). Immunostaining for SARS-CoV-2, spike proteins. Original magnification 200×. (**b**) Presence of viral spike proteins in the eccrine sweat glands (brown stain). Immunostaining for SARS-CoV-2, spike proteins. Original magnification 400×.

Immunohistochemical analysis was done in all three cases using the SARS-CoV-2 anti-spike proteins antibody, yielding positive results in the eccrine sweat glands (Figure 3.

**Figure 4:**
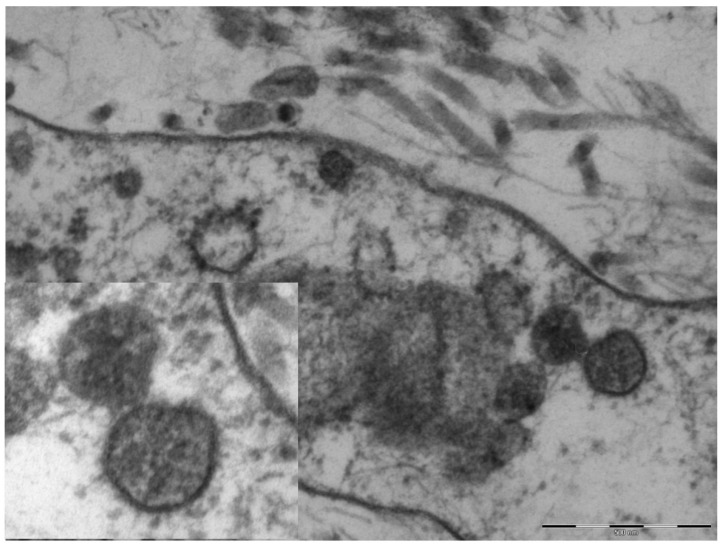
Original magnification 89,000× fibroblast cytoplasm containing numerous maturing coronaviruses. Fragmented collagen fibers are visible in the interstice. In the box, 110,000×, whole virions are evident with spikes of different lengths and an electron-dense peripheral rim.

An electron microscopy study was performed in only one case, showing marked hypertrophy of the endothelial cells, the nuclei being characterized by finely distributed chromatin and retiform nucleoli. The cytoplasm contained very few organelles, with few small globular mitochondria. The fibroblasts were remarkably elongated and surrounded by very short collagen fibers. Spherical structures ranging from 65 to 136 nm were visible in the cytoplasm of endothelial cells and fibroblasts (Figure 4), characterized by a peripheral electron-dense rim and a clearer core. These structures were more frequently near the cell membrane or externally attached to it. Aggregates of multiple viral particles were grouped together close to phagosome-like vesicles. In some images, faintly electron-dense projections of different sizes (6–10 nm)—corresponding to the virus spikes—were visible, depicting a solar crown.

## 4. Discussion

The nodular lesions described in the present report differ from the chilblain-like lesions related to COVID-19 due to their frankly nodular appearance and chronic course. However, they recall the chilblain-like lesions that were reported with increasing frequency during the pandemic period [18,19], in regards to the localization on the feet, the lack of respiratory manifestations and negative molecular tests, histological aspects of vasculitis and small vessels thrombotic phenomena, and above all the presence of glyco-spike proteins in endothelial cells [20,21,22]. These had been detected in previous studies of chilblain-like lesions [12], both in endothelial cells and in eccrine sweat glands, similar to those described in the present report. There is still considerable debate on the use of electron microscopy in the course of COVID-19 infection, as the initial confirmations of SARS-CoV-2 in chilblain-like lesions [12] were subsequently disavowed [23]. This method seems to provide further proof in the presence of other, more credible elements, rather than offering proof in itself. In any case, epidemiologic data allow us to hypothesize that both acute erythematous-edematous chilblain-like lesions and chronic nodular lesions reminiscent of deep granuloma annulare may be the expression of Coronavirus contagion in young subjects who are particularly reactive to the virus. Some authors have recently demonstrated the presence of Myxovirus resistance protein A, a recognized tissue marker of interferon 1 activity, in the epidermis, endothelial cells, and inflammatory infiltrate of cutaneous chilblain-like lesions [23,24,25]. This finding supports the hypothesis that in young subjects with an energetic interferon type 1 anti-virus response, the latter can on the one hand neutralize or minimize the symptoms of viral infection and justify the negativity of the molecular tests, and on the other hand, as a side effect, induce microangiopathy, as occurs in genetic interferonopathies and in systemic lupus erythematosus.

## 5. Conclusions

Overall, we describe a hybrid pattern of dermatologic lesions likely related to SARS-CoV-2, confirmed only by EM and immunohistochemistry assay. In our opinion, the acral nodular lesions described herein may be related more to immune responses secondary to the viral infection than to the direct cytopathic effect of the virus. Many varied skin manifestations have been described in these months of the pandemic, sometimes not correlated with histopathological and immunohistochemical data. A careful evaluation of the time lapse since the presumed contact with SARS-CoV-2 is of fundamental importance to reconstruct the clinical history of patients with COVID-related skin manifestations. Further studies are needed to fully explore this issue and also to gain a more in-depth understanding of the spectrum of skin manifestations that can be caused by SARS-CoV-2.

## Data Availability

This does not applicable.
